# Now you see it, now you don't: Flanker presence induces the word concreteness effect

**DOI:** 10.1016/j.cognition.2021.104945

**Published:** 2022-01

**Authors:** Aaron Vandendaele, Jonathan Grainger

**Affiliations:** a Department of Experimental Psychology, Ghent University, Belgium; b Laboratoire de Psychologie Cognitive, CNRS & Aix-Marseille University, France; c Institute for Language, Communication and the Brain, Aix-Marseille University, France

**Keywords:** Flankers task, Word concreteness, Lexical decision, Depth-of-processing

## Abstract

Can the presence of unrelated flanker words change the way that lexical decisions are made to target words in the flankers task? Here we examined the impact of flanker presence on the effects of word concreteness. Target words had high or low concreteness ratings (e.g., *fork*, *free*) and were either presented in isolation or flanked to the left and right by an unrelated word (e.g., *cold free cold*) that was irrelevant for the task. Results revealed that the facilitatory effect of concreteness (faster responses to concrete words compared with abstract words) was significantly greater in the presence of flankers. A control experiment revealed the same pattern with pseudoword and nonword flankers. We conclude that the mere presence of flanking letter strings causes a greater depth of processing of target words. We further speculate that this might arise by flankers inducing a more “sentence-like” context by the presence of multiple, spatially distinct letter strings, that prohibits the use of more superficial decision processes and can be used to make lexical decisions to isolated words.

## 1 Introduction

Several recent studies have adopted the flanker paradigm as a means to bridge the gap between the two relatively independent lines of research on single-word reading on the one hand and sentence reading on the other (see Snell & Grainger, 2019, for an overview). In this version of the flanker task, participants respond to central target words that are flanked to the left and to the right by stimuli that are irrelevant for the task. The flanking stimuli can be related to targets on a given dimension of interest, or unrelated to targets. The seminal work of Dare and Shillcock (2013) revealed effects of orthographic relatedness when the task is lexical decision (see also Bonin, Méot, & Bugaiska, 2018; Grainger, Mathôt, & Vitu, 2014; Snell, Bertrand, & Grainger, 2018; Snell, Bertrand, Meeter, & Grainger, 2018; Snell, Declerck, & Grainger, 2018; Snell, Mathôt, Mirault, & Grainger, 2018; Snell, Meeter, & Grainger, 2017; Snell, van Leipsig, Grainger, & Meeter, 2018; Snell, Vitu, & Grainger, 2017). Morphological relatedness also impacts on lexical decisions to target words (Grainger, Snell, & Beyersmann, 2020), but not phonological relatedness (Cauchi, Lété, & Grainger, 2020). When the task involves a syntactically- or semantically-based decision (e.g., noun vs. verb; animate vs. inanimate) then the syntactic or semantic compatibility of flanker words is found to impact on performance (Snell, Declerck, & Grainger, 2018; Snell, Meeter, & Grainger, 2017). The results of this line of research were foundational with respect to the development of a theoretical framework that integrates word identification processes in an account of sentence-level processing and specifies how different word identities can be simultaneously mapped onto distinct spatiotopic locations during reading (Snell, Meeter, & Grainger, 2017; Snell, van Leipsig, Grainger, & Meeter, 2018).

In the present study, we leave aside the question of effects of flanker relatedness in order to address a central postulate of the above-mentioned theoretical framework – that the flankers task can provide an interesting new window on how words are identified when presented in a multiple-word context.^1^ One piece of evidence in favor of this postulate is that the distribution of spatial attention in the flankers task mimics that found in natural reading – that is, there is a bias in the direction of reading (Snell & Grainger, 2018; Snell, Mathôt, Mirault, & Grainger, 2018). However, the key and quite surprising evidence found so far was the demonstration that the effect of a given variable on lexical decisions to a word target changes as a function of whether or not the word is surrounded by unrelated flanker words (Meade, Grainger, & Declerck, 2020). The manipulation in the Meade et al. study was a classic manipulation of the number of orthographic neighbors of target words (single letter substitution neighbors), referred to as neighborhood density. Effects of neighborhood density interacted with the manipulation of flanker presence, with the inhibitory effect (high density targets were harder to respond to than low density targets) being greater in the presence of flankers. This inhibitory pattern has been reported in studies using perceptual identification tasks (e.g., Carreiras et al., 1997; Grainger & Jacobs, 1996) or in silent reading for meaning (Pollatsek et al., 1999), whereas the effect is typically facilitatory in single word lexical decisions (e.g., Andrews, 1989).^2^

Here we build on this remarkable finding, and the interpretation offered by Meade et al. (2021). According to Meade et al., the presence of unrelated word flankers induces a change in the processes used to make a lexical decision. In the model proposed by Grainger and Jacobs (1996), there are two mechanisms that can be applied to make a “word” decision – either by identifying the word, or by using a measure of global lexical activity (i.e., the summed activity of all activated words). It is the second mechanism that produces facilitatory effects of neighborhood density in that model. Meade et al. therefore conjectured that the presence of flankers caused participants to abandon use of this mechanism, and to base their “word” decisions on word identification. Why would the presence of flankers induce such a change? The hypothesis entertained by Meade et al., and that forms the starting point of the present work, is that flankers induce a more “sentence-like” reading like behavior, and that during sentence reading words must be identified in order to recover semantic and syntactic information for sentence comprehension. This does not imply that participants were actually reading the three unrelated words as if they were reading a sentence, but simply that the multiple-word context induced by flankers caused a change in the way participants made their lexical decisions to central targets.

In the present study we tested the hypothesis that the word identification strategy for making lexical decisions induced by flanker presence should lead to stronger effects of a semantic variable. To do so, we manipulated the concreteness of target words, and the effect of this manipulation was examined in the presence or absence of unrelated flanker words. We predicted that concreteness effects on lexical decisions would be greater in the presence of flanking words. Although initial investigations revealed facilitatory effects of concreteness (higher performance to concrete compared with abstract words) in lexical decisions to isolated words (Kroll & Merves, 1986^3^; Schwanenflugel & Shoben, 1983), subsequent research on the effects of concreteness has revealed mixed findings as a function of type of sentence context (highly constraining or neutral: e.g., Schwanenflugel & Stowe, 1989), dependent variable (behavioral or electrophysiological: e.g., Barber, Otten, Kousta, & Vigliocco, 2013), and whether or not possible confounding factors are controlled for (e.g., Kousta, Vigliocco, Vinson, Andrews, & Del Campo, 2011). Nevertheless, the consensus that has emerged from this research is that concrete words elicit more semantic processing than abstract words. The clearest evidence for this comes from studies showing greater N400 amplitudes to concrete words compared with abstract words (Barber et al., 2013; Dufau, Grainger, Midgley, & Holcomb, 2015; West & Holcomb, 2000). Barber et al. (2013) suggested that this finding can be reconciled with null effects (and even reversed effects) of concreteness seen in the lexical decision task by assuming that lexical decisions to single words can be based on more superficial information, whereas the N400 would provide a better reflection of semantic processing. Here we sought evidence for a semantic influence on lexical decisions when these are made in the context of a sequence of words.

## 2 Methods

### 2.1 Participants

Eighty-two participants ranging from 18 to 39 years old (55 female; mean age = 23.39, *SD* = 4.83) volunteered for this experiment. The data were collected online, and the experiment was made available for one month and a half. Participants were either recruited through announcements spread by the French Information Network for Cognitive Sciences (RISC) or through various social media platforms. Each participant indicated being a French native speaker.

### 2.2 Materials and design

Sixty abstract (e.g., *rich*, *end*, *myth*, *south*) and 60 concrete (e.g., *nail, razor, coat, wall*) French target words were chosen from a recently developed French norming database (Bonin et al., 2018).^4^ We selected target words from the extremes of the concrete – abstract continuum while respecting the constraints imposed by the other variables we controlled for. This led to concreteness being best expressed as a binary variable. Using the French Lexicon Project database (Ferrand et al., 2010), our targets were controlled for length, word frequency, orthographic neighborhood density, context availability, valence and arousal. Target and flanker frequencies are expressed in Zipf values (see van Heuven, Mandera, Keuleers, & Brysbaert, 2014); word length in number of letters; neighborhood density (the number of single-letter substitution neighbors); and the remaining variables were measured using a 5-point Likert scale. We employed two-tailed *t*-tests to check for significant differences between the concrete and abstract word categories, and these tests revealed that the only significant difference was for concreteness itself (see Table 1). 120 pseudoword targets were selected using the Wuggy pseudoword generator (Keuleers & Brysbaert, 2010), and were matched to the word targets on length and sub-syllabic structure. Each target word was paired with a flanker word that was matched on length and frequency and did not have any orthographic or semantic overlap^5^ with the target word. Each target word was presented in an isolated target condition and a condition where is was surrounded to the left and to the right by an unrelated flanker word. This represents the factor ‘flanker presence’ which was crossed with concreteness (concrete vs. abstract words) in a 2 × 2 factorial. A blocked design was used in which participants could either receive the isolated target condition or the flanked target condition first, with the order of blocks was counterbalanced across participants. Flanker presence was manipulated using a Latin-square design such that each target word was seen in both the flanker and the isolated condition, but only once per participant.

**Table 1:** Overview of the stimuli properties.

	Concrete	Abstract	*t*-value
Word length	4.62 (0.96)	4.83 (0.96)	*t*(118) = −1.24
Target frequency^a^	4.27 (0.46)	4.35 (0.76)	*t*(118) = −0.74
Flanker frequency^a^	4.23 (0.44)	4.30 (0.74)	*t*(118) = −0.59
Target concreteness^b^	4.36 (0.33)	2.02 (0.23)	*t*(118) = **45.42**
Context availability^b^	3.16 (0.37)	3.10 (0.58)	*t*(118) = 0.77
Valence^b^	3.07 (0.58)	3.15 (1.07)	*t*(118) = −0.58
Arousal^b^	2.76 (0.53)	2.85 (0.73)	*t*(118) = −0.79
Neighborhood density^c^	7.48 (4.18)	6.15 (4.20)	*t*(118) = 1.74^d^

Note. Values between parentheses indicate standard deviations. Significant t-values in bold.

a The French Lexicon Project (Ferrand et al., 2010).

b Bonin et al. (2018).

c CLEARPOND data base (Marian, Bartolotti, Chabal, & Shook, 2012).

d We note the marginally significant difference in neighborhood density. However, given the results of Meade et al. (2021), this should lead to a stronger inhibitory effect of concreteness in the presence of flankers, hence countering our prediction.

### 2.3 Apparatus

The stimuli and experimental design were implemented using OpenSesame (Mathôt, Schreij, & Theeuwes, 2012) and imported through the OSWeb extension into JATOS (Lange, Kühn, & Filevich, 2015). Stimuli were presented in lowercase using a 30-point monospaced font (droid sans mono, the standard in OpenSesame). Participants were instructed to use their personal computers and sit 50 cm from their screen so that each character space subtended 0.53 degrees of visual angle.

### 2.4 Procedure

**Fig. 1:**
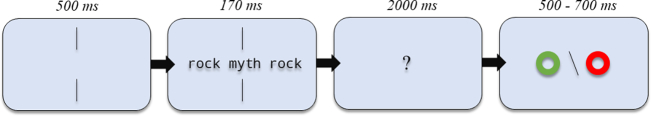
Example of the procedure used with a flanked abstract word. The examples are given in English for convenience.

Before the experiment began, participants received on-screen instructions as a function of which block they were assigned to first (single words or flankers). Within each block the concrete and abstract target words were randomly intermixed. Each trial began with two vertically aligned fixation bars that stayed on-screen for a duration of 500 ms. Afterwards, the stimuli appeared on screen for 170 ms, after which the participant had 2000 ms to give a response. Depending on this response, a green (correct) or red (incorrect) dot would appear for a random duration between 500 and 700 ms. After this, a new trial would begin (see Fig. 1 for a summary of the procedure). The experiment consisted out of 2 blocks, each containing 120 trials. Between the blocks, a break was provided until participants indicated that they were ready to continue. Before the actual experiment began, participants received 12 practice trials containing examples (not shown in the main experiment) of all conditions. Responses were given with an azerty keyboard, using the ‘q’-button to indicate a nonword and the ‘m’-button to indicate a word. On average, the experiment lasted about 15 min. In total, we had 2460 observations per condition, exceeding the recommended 1600 for abundant statistical power by Brysbaert and Stevens (2018). Moreover, the work of Meade et al. (2021), which used the same design as this study, observed a significant interaction effect with 1680 observations.

## 3 Results

**Fig. 2:**
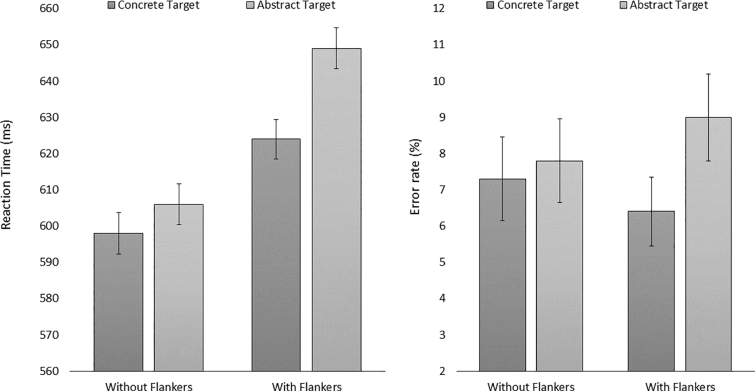
Mean RTs (in milliseconds) and error rates (percentages) per condition. Error bars indicate 95% confidence intervals.

Means of reaction times (RTs) and error rates per condition are shown in Fig. 2. The data were analyzed using linear mixed effect models fitted with the (g)lmer functions from the lme4 package (Bates, Mächler, Bolker, & Walker, 2015) in RStudio version 3.6.1 statistical computing environment. Items and participants were entered as crossed random effects, and where the model structure allowed it, by-item and by-participant random intercepts were included (Baayen, Davidson, & Bates, 2008; Barr, Levy, Scheepers, & Tily, 2013). We report *b*-values, standard errors (*SE*s) and *t*- or *z*-values (for RTs and error rates respectively), with those beyond |1.96| deemed as significant (*b*-values and *SE*s were multiplied by a fixed factor to increase interpretability). Only data from trails with word targets were included in these analyses.^6^ For the analysis of RTs, only correctly answered trials were included, leading to the exclusion of 7.24% of the observations. Furthermore, trials which exceeded the 2.5 SD interval from the grand mean were also excluded (2.68%). In order to meet the model's assumption that the data are distributed normally, a logarithmic transformation (Log^10^(RT)) was performed prior to the analysis. The condition without flankers and the concrete word category were always used as the reference level. In order to account for possible effects of block order, each model included a predictor indicating if participants saw the isolated target or the flanker condition first.

### 3.1 RT analyses

In the RT analysis there was a main effect of concreteness (*b* = 1.59, *SE* = 0.48, *t* = 3.31), and a main effect of flanker presence (*b* = 3.11, *SE* = 0.27, *t* = 11.4). Concreteness facilitated lexical decisions whereas flanker presence caused interference. Crucially, there was a significant interaction effect between concreteness and flanker presence (*b* = −1.44, *SE* = 0.40, *t* = −3.62), with a greater facilitatory effect of concreteness being found in the presence of flankers (see Fig. 2).^7^ Block order did not affect performance (*b* = −1.03, *SE* = 1.32, *t* = −0.78) and did not interact with concreteness (*b* = −0.85, *SE* = 0.44, *t* = −1.93) or flanker presence (*b* = −0.07, *SE* = 0.43, *t* = −0.17). The three-way interaction was not significant (*b* = 0.93, *SE* = 0.65, *t* = 1.51).^8^

### 3.2 Error analyses

The analysis of errors revealed a similar numerical trend (see Fig. 2) but there were no significant effects (concreteness: *b* = −0.09, *SE* = 0.20, *z* = −0.46; flanker presence: *b* = −0.22, *SE* = 0.13, *z* = −1.67; concreteness X flanker interaction: *b* = 0.24, *SE* = 0.20, *z* = 1.19). As with RTs, there was no main effect of block order (*b* = 0.24, *SE* = 0.22, *z* = 1.09) and no interactions with this factor (concreteness: *b* = −0.10, *SE* = 0.23, *z* = −0.45, flanker presence: *b* = 0.06, *SE* = 0.22, *z* = 0.26, three-way interaction: *b* = 0.29, *SE* = 0.33, *z* = 0.87).

## 4 Control experiment with pseudoword and nonword flankers

In order to be sure that the effects of flanker presence found in the main experiment were not being driven by uncontrolled relations between target and flanker words such as target-flanker congruency (see e.g., Snell, Meeter, & Grainger, 2017, Snell, Vitu, & Grainger, 2017, for an effect of syntactic category congruency in the flankers task), we ran a second experiment where flankers could either be pseudowords (e.g., *flink*) or random letter strings (e.g., *sldmf*). Results showed that the effect of concreteness was still present with these non-lexical flankers in both RTs (*b* = 1.17, *SE* = 0.58, *t* = 2.02) and error rates (*b* = −0.11, *SE* = 0.04, *z* = −2.19). This clearly demonstrates that uncontrolled lexical relations between targets and flankers were unlikely to be the source of the concreteness effects seen in the main experiment.^9^

## 5 Discussion

Building on the work of Meade et al. (2021), the present study provided a further investigation of how the mere presence of flanker words that are unrelated in any way to central target words (e.g., *cold free cold*) causes a greater depth of processing of the targets compared with an isolated target condition (e.g., *free*). Meade et al. found that flanker presence led to greater inhibitory effects of orthographic neighborhood density on lexical decisions to target words. They concluded that the presence of flankers encourages participants to use word identification to trigger a “word” response in the lexical decision task as opposed to a more superficial response strategy that could be applied when processing isolated words, based, for example, on global lexical activity (Grainger & Jacobs, 1996). This result provided support for the hypothesis that the presence of flanker words stimulates a more “sentence-like” processing of target stimuli, and therefore encourages the use of a word identification strategy to make “word” responses. The central idea is that the presence of a series of spatially defined letter strings, as is the case in sentence reading, triggers the kind of processing of target words that is necessary for sentence comprehension (i.e., word identification and retrieval of semantic and syntactic information). We therefore predicted that effects of word concreteness, a semantic variable, would be greater in the presence of flankers. As predicted, we found that the facilitatory effect of concreteness (faster RTs to concrete words compared with abstract words) was significantly greater in the presence of flanker words, compared with isolated targets.

The present results add to the growing evidence that the reading version of the flankers task, with horizontally aligned target and flanker words, encourages a reading-for-meaning style of processing that resembles the word identification processes operating during sentence reading. Note that this does not necessarily imply that participants are attempting to compute a sentence-level representation of the sequence of target and flanker words, and indeed our own findings suggest that this is not the case (Snell, Meeter, & Grainger, 2017; Snell, Vitu, & Grainger, 2017; Vandendaele, Declerck, Grainger, & Snell, 2020). We would argue that the greater depth of processing of target words in the presence of flankers is due to the mere presence of letter strings that are horizontally aligned with the target, and which visually simulate a sentence-like structure.

The results of our control experiment demonstrate that flanking stimuli do not need to be real words in order to trigger this shift in processing strategy. Crucially, it could be argued that the effects obtained in the main experiment are simply due to flankers inducing a more careful mode of processing, with participants making sure that they respond to the central target and not to flankers. We would argue that greater care would be required with word flankers compared with nonword flankers. Furthermore, the results obtained with pseudoword and nonword flankers suggests that the lexical characteristics of flanker words should not modulate their impact on target word processing. Future research could examine whether this is indeed the case by manipulating such lexical characteristics as flanker frequency or flanker concreteness. Another crucial experiment for future work would to examine whether flankers have to be formed of letters or whether any kind of non-alphabetic flanking stimulus would generate the same pattern. Our prediction is that it is the alphabetic nature of flankers that is key to obtaining the present findings.^10^

The present results also confirm the difficulty in replicating early observations of word concreteness effects on lexical decisions to isolated words (Kroll & Merves, 1986; Schwanenflugel & Shoben, 1983), with more recent studies showing either a null effect (see Holcomb, Kounios, Anderson, & West, 1999) or even a reversed effect (i.e., faster responses to abstract words: Barber et al., 2013; Kousta et al., 2011).^11^ Crucially, we demonstrate that the exact same set of stimuli do show a very robust facilitatory effect of concreteness when presented with unrelated flanker words. This finding aligns nicely with the results of Barber et al. (2013) who found a significant increase in N400 amplitude to concrete words compared with abstract words, and a reversed effect of concreteness on lexical decision RTs. Following Barber et al., we would argue that lexical decisions to isolated words can be made on the basis of relatively superficial information (i.e., shallow processing), whereas N400 amplitude is sensitive to lexical semantics, and the flankers task encourages participants to use less superficial information (e.g., word identification, semantics) when making a lexical decision.

Finally, might it be possible that the pattern of effects reported here is simply due to the presence of flanker stimuli slowing the processing of target words via, for example, changes in the distribution of visual attention (e.g., Eriksen & Eriksen, 1974), or interference caused by the spatial pooling of information across target and flankers (i.e., crowding: Pelli & Tillman, 2008), or decision-level interference (e.g., Rouder & King, 2003).^12^ It is certainly clear that flankers did produce an overall interference in the processing of both word and pseudoword targets in the present study, and it is possible that the slower processing of target words could leave more room for concreteness effects to emerge. Although we cannot completely rule-out this alternative interpretation, in Appendix C we show that the greater effects of concreteness in the presence of flankers remains relatively constant across quantiles, even although the overall effect of concreteness does increase as average RT increases.

## 6 Conclusions

The present study demonstrated that the mere presence of unrelated flanking letters leads to a greater facilitatory effect of concreteness (faster lexical decisions to concrete than to abstract words) compared with the effect obtained with isolated targets. We interpret this finding as reflecting a greater depth of processing of target words in the presence of flankers, and notably that the presence of flanking letters encourages participants to make their “word” decision on the basis of word identification. Although flanker presence does induce overall longer RTs, and greater average RTs lead to an increase in concreteness effects with or without flankers, we argue that flanker presence induces a change in depth of processing that operates in addition to any impact of the overall interfering effect of flankers.

## Open practice statement

All materials and data can be found on OSF through the following link: https://osf.io/ysb7v/

## Declaration of Competing Interest

None.

## Figures and Tables

**Table A1:** *b*-, *t*-values and standard errors of the reaction time analysis.

Factors	*b*-value	SE	*t*-value
Concreteness	1.59	0.48	**3.11**
Flankers	3.11	0.27	**11.4**
Block order	−1.03	1.32	−0.78
Concreteness * Flankers	−1.44	0.4	**−3.62**
Concreteness * Block order	−0.85	0.44	−1.93
Flankers * Block order	−0.07	0.43	−0.17
Concreteness * Flankers * Block order	0.93	0.62	1.51

**Table A2:** *b*-, *z*-values and standard errors of the error rate analysis.

Factors	*b*-value	SE	*z*-value
Concreteness	−0.09	0.2	−0.46
Flankers	−0.22	0.13	−1.67
Block order	0.24	0.22	1.09
Concreteness * Flankers	0.24	0.2	1.19
Concreteness * Block order	−0.1	0.23	−0.45
Flankers * Block order	0.06	0.22	0.26
Concreteness * Flankers * Block order	0.29	0.33	0.87

**Table B1:** Condition means for the effect of Flanker presence on responses to pseudowords targets.

Factors	RTs			Error rates		
	Mean	SD	95% CI	Mean	SD	95% CI
With flanker	669	135	[652–687]	29.67	4.57	[25.62–33.72]
Without flanker	652	133	[642–661]	7.89	2.69	[6.52–9.26]

**Table B2:** *b*-, *t*-values and standard errors of the reaction time analysis.

Factors	*b*-value	SE	*t*-value
Flanker presence	1.73	0.27	**7.63**
Block order	−0.18	1.34	−0.14
Flankers * Block order	1.19	0.36	**3.36**

**Table B3:** *b*-, *z*-values and standard errors of the error rate analysis.

Factors	*b*-value	SE	*z*-value
Flanker presence	2.07	0.11	**18.4**
Block order	0.36	0.41	0.88
Flankers * Block order	0.06	0.17	0.34

**Table C1:** LME output for raw data, for data after 2.5 SD exclusion and for the final dataset.

	Raw			After 2.5 SD excl.			After Log-transform		
	*b*	*SE*	*t*	*b*	*SE*	*t*	*b*	*SE*	*t*
Concreteness	32.13	8.83	**3.64**	26.11	7.46	**3.50**	1.59	0.48	**3.31**
Flankers	54.45	5.52	**9.85**	46.89	4.14	**11.33**	3.11	0.27	**11.40**
Con x Flankers	−24.0	8.12	**−2.96**	−23.2	6.07	**−3.82**	−1.44	0.40	**−3.62**

**Fig. C1:**
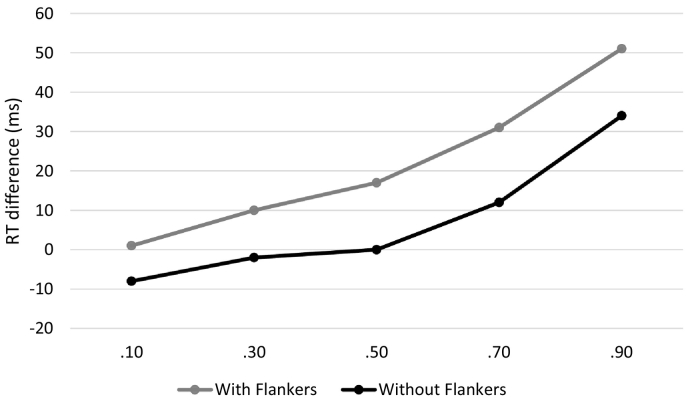
Delta plots of concreteness effects (RT differences between concrete and abstract target words) in the main Experiment for 0.1, 0.3, 0.5, 0.7, and 0.9 quantiles.

## Appendix A Results of LME and GLME analyses for word targets in the main experiment

Significant *t*-values are indicated in bold.

## Appendix B Results of LME and GLME analyses for pseudoword targets in the main experiment

Significant t-values are indicated in bold.

## Appendix D Supplementary data

Supplementary materials Image 1

## Footnotes

1 We note here that the key characteristic of the reading version of the flankers task, compared with priming manipulations that also involve multiple stimuli, is the simultaneous presentation of spatially distinct and horizontally aligned stimuli (see Snell, Bertrand, Meeter, & Grainger, 2018), for a comparison of results obtained with priming and flanker procedures).

2 We also note the converging evidence from a study that revealed weak correlations between isolated lexical decision latencies and eye movement measures of word-in-text processing (Dirix et al., 2019).

3 The facilitatory effect of concreteness in the Kroll and Merves study was mainly evident when a block of abstract words followed a block of concrete words. This points to possible strategic factors at play. In the present study we randomly intermixed the concrete and abstract target words.

4 We note that concreteness correlates highly with imaginability (*r* = 0.89 in the Bonin et al., 2018, database). However, given that imaginability is as closely related to semantic processing as concreteness, we can ignore this correlation for the present purposes.

5 In order to fully control for semantic overlap across target-flanker pairs, we ran a separate rating study. Fifty participants were asked to rate how semantically similar the word pairs were on a scale from 1 to 5. We also included 80 highly semantically-related word pairs from the Lakhzoum, Izaute, and Ferrand (2020) database in order to induce a full use of the rating scale. Results showed that ratings between target-flanker pairs were overall very low (M = 1.23, SD = 0.15, Min = 1.02, Max = 1.78). A two-tailed *t*-test revealed that these ratings did not differ significantly for the concrete and abstract target words *t*(118) = 0.82, *p* = 0.41.

6 Following the request of a reviewer we provide the analyses of pseudoword targets in Appendix B.

7 It is interesting to note that non-significant +8 ms effect of concreteness seen in the isolated target condition (*b* = 0.13, *SE* = 0.08, *t* = 1.74) aligns nicely with the effect obtained with the same stimuli in the French Lexicon Project (Ferrand et al., 2010), which rendered a non-significant +7 ms effect (*t*(118) = 0.66).

8 Following the recommendation of a reviewer, and the evidence that log transformation can modify effect patterns (e.g., Yap, Balota, & Tan, 2013) we also performed the same analysis on the RTs without using the 2.5SD exclusion criteria and without a log-transformation. The same pattern was found with all three types of preprocessing (see Appendix C).

9 A full description of this experiment can be found in the supplementary materials.

10 We note that in unpublished work manipulating the position of related word flankers (left or right of the target), we found a significant difference between unrelated word flankers and flankers composed of hash marks (#####) located at the opposite location to the related flankers, with greater interference caused by the unrelated words.

11 We again point out that the concrete and abstract word targets tested in the present study render an almost identical null effect when their means are extracted from the French Lexicon Project (i.e., isolated lexical decisions: Ferrand et al., 2010).

12 We note that the interfering effect of pseudoword flankers compared with nonword flankers on decisions made to pseudoword targets in our control experiment (see Supplementary Materials) could well be a decision-level effect.
