# Supplementary material for: Now you see it, now you don't: Flanker presence induces the word concreteness effect
Source: Cognition. 2022 Jan;218:104945. doi: 10.1016/j.cognition.2021.104945 (PMC8655615; doi:10.1016/j.cognition.2021.104945)
Supplement: Supplementary file 1 — Supplementary materials [file mmc1.docx]

**Supplementary materials: The pseudoword and nonword flanker control experiment.**

This document is a supplement to Vandendaele & Grainger, *Now you see it, now you don’t: Flanker presence induces the word concreteness effect*

Below we report the results of the pseudoword and nonword flanker version of the main experiment. Sixty participants (44 female, mean age = 23.3, SD = 6.5) took part in this study. The software, implementation and analyses were identical to the main experiment. The only change was in terms of methodology: the same set of concrete and abstract target words were used, but these target words could either be flanked by a pseudoword (e.g., *flink*) or by a nonword letter string (e.g., *sldmf*). This new factor ‘flanker category’ thus replaced the factor ‘flanker presence’ from the main experiment. Furthermore, the blocked presentation was also removed (i.e., pseudo- and nonword flankers were presented randomly). The rest of the experiment remained the same (see Figure S1 for the procedure).

Pseudowords were selected from the same French Lexicon Project database (Ferrand et al, 2010). The main criteria for both pseudo- and nonwords were that they had to have no orthographic overlap with the corresponding target word. Materials and data can be found on OSF through the same link as the main experiment: <https://osf.io/ysb7v/>


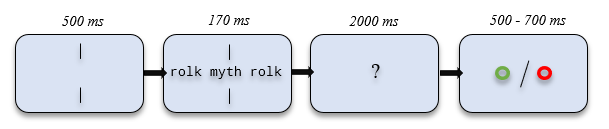


*500 ms*

*170 ms*

*500 - 700 ms*

*2000 ms*

?

rolk myth rolk

**Figure S1.** Example of the procedure used with an abstract word and pseudowords as flankers. The examples are given in English for convenience.

**Results**

Word targets

The results are summarized in Figure S2. For the analyses of RT, only correctly answered trials were included, which led to the exclusion of 6.65% of the observations. Furthermore, trials that exceeded the 2.5SD interval from the grand mean were also excluded (1.99%). Data were log-transformed (LOG^10^(RT)) to meet the assumption of normal distribution.

Overall, the concreteness effect was still present in the RT-data when target words were flanked with pseudowords and nonwords (*b* = 1.17, *SE* = 0.58, *t* = 2.02). There was no interaction between flanker type and target category (*b* = 0.02, *SE* = 0.61, *t* = 0.27) and no effect of flanker type (*b* = -0.2, *SE* = 0.43, *t* = -0.56). A similar numerical trend was observed in the error rates, with a main effect of concreteness (*b* = -0.11, *SE* = 0.04, *z* = -2.19) and no effect of flanker type (*b* = -0.01, *SE* = 0.03, *z* = -0.52). Note here that the most parsimonious model for the error rates did not include the interaction effect. Condition means are shown in Figure S2.

**Figure S2.** Mean RTs (in milliseconds) and error rates (percentages) per condition. Error bars indicate 95% confidence intervals.

Pseudoword targets

The data were analyzed in the same way as for word targets. Condition means are shown in Table S1. RTs to pseudoword targets were significantly slower when the flankers were pseudowords compared with nonword flankers (*b* = 0.15, *SE* = 0.04, *t* = 4.09). Error rates did not differ significantly as a function of Flanker type (*b* = -10, *SE* = 0.12, *z* = -0.87).

|  | RTs | | | Error rates | | |
| --- | --- | --- | --- | --- | --- | --- |
| Factors | Mean | SD | 95% CI | Mean | SD | 95% CI |
| Pseudoword | 727 | 163 | [704 - 749] | 10.94 | 3.12 | [9.02 – 12.86] |
| Nonword | 709 | 158 | [692 - 726] | 10.26 | 3.02 | [8.53 – 11.99] |

**Table S1.** *Condition means for the effect of Flanker type on responses to pseudoword targets*
